# Inflammation Subverts Hippocampal Synaptic Plasticity in Experimental Multiple Sclerosis

**DOI:** 10.1371/journal.pone.0054666

**Published:** 2013-01-23

**Authors:** Robert Nisticò, Dalila Mango, Georgia Mandolesi, Sonia Piccinin, Nicola Berretta, Marco Pignatelli, Marco Feligioni, Alessandra Musella, Antonietta Gentile, Francesco Mori, Giorgio Bernardi, Ferdinando Nicoletti, Nicola B. Mercuri, Diego Centonze

**Affiliations:** 1 IRCSS Fondazione Santa Lucia, Rome, Italy; 2 Dipartimento di Fisiologia e Farmacologia, Università di Roma "La Sapienza", Rome, Italy; 3 Clinica Neurologica, Dipartimento di Medicina dei Sistemi, Università Tor Vergata, Rome, Italy; 4 Laboratorio di Farmacologia della Plasticità Sinaptica, EBRI-European Brain Research Institute, Rome, Italy; 5 IRCCS Neuromed, Pozzilli, Italy; University Hospital of Heidelberg, Germany

## Abstract

Abnormal use-dependent synaptic plasticity is universally accepted as the main physiological correlate of memory deficits in neurodegenerative disorders. It is unclear whether synaptic plasticity deficits take place during neuroinflammatory diseases, such as multiple sclerosis (MS) and its mouse model, experimental autoimmune encephalomyelitis (EAE). In EAE mice, we found significant alterations of synaptic plasticity rules in the hippocampus. When compared to control mice, in fact, hippocampal long-term potentiation (LTP) induction was favored over long-term depression (LTD) in EAE, as shown by a significant rightward shift in the frequency–synaptic response function. Notably, LTP induction was also enhanced in hippocampal slices from control mice following interleukin-1β (IL-1β) perfusion, and both EAE and IL-1β inhibited GABAergic spontaneous inhibitory postsynaptic currents (sIPSC) without affecting glutamatergic transmission and AMPA/NMDA ratio. EAE was also associated with selective loss of GABAergic interneurons and with reduced gamma-frequency oscillations in the CA1 region of the hippocampus. Finally, we provided evidence that microglial activation in the EAE hippocampus was associated with IL-1β expression, and hippocampal slices from control mice incubated with activated microglia displayed alterations of GABAergic transmission similar to those seen in EAE brains, through a mechanism dependent on enhanced IL-1β signaling. These data may yield novel insights into the basis of cognitive deficits in EAE and possibly of MS.

## Introduction

Learning and memory processes depend on the ability of brain circuitries to retain information in the form of enduring use-dependent changes of synaptic strength [1]. Both long-term potentiation (LTP) and long-term depression (LTD) of excitatory synaptic transmission can be induced experimentally at a same synapse in response to different patterns of repetitive synaptic activation [2], [3]. The ability of synapses to undergo either LTP or LTD increases information storage capability and ensures optimal circuit flexibility, which is essential for higher cognitive abilities [4].

Multiple sclerosis (MS), a neuroinflammatory disorder characterized by demyelination and progressive axonal loss, is associated with early cognitive deficit, which has a significant impact on the quality of life of patients [5]. Recent studies highlight the importance of inflammation-induced synaptic dysfunction in the very early phases of MS [6]–[8]. This raises the possibility that inflammatory molecules secreted by autoreactive lymphocytes or activated microglia in the CNS interfere with physiological mechanisms of synaptic plasticity leading to early cognitive dysfunction in MS.

To shed some light on the relationship between neuroinflammation and cognitive impairment, here we studied hippocampal synaptic plasticity and transmission in experimental autoimmune encephalomyelitis (EAE), which models MS in mice. We also explored the role of activated microglia and of the pro-inflammatory cytokine interleukin-1β (IL-1β) on neurotransmission, neuronal integrity, synaptic plasticity and network activity in this neuroinflammatory disorder.

Our results showed that LTP appearance was favored over LTD in response to repetitive synaptic activation in EAE mice, and that IL-1β secreted by activated microglia played a crucial role in this alteration by interfering with GABAergic synapses in the hippocampus. Importantly, we also demonstrate that the impairment of inhibitory neurotransmission was associated with a selective loss of parvalbumin (PV)-positive GABAergic neurons and with reduced gamma oscillations in the hippocampus of EAE mice.

## Materials and Methods

### Ethics Statement

All efforts were made to minimize animal suffering and to reduce their number, in accordance with the European Community Council Directive of 24 November 1986 (86/609/EEC) and approved by the Ethical Committee on animal experiments of Santa Lucia Foundation (Rome, Italy).

### EAE Induction

EAE was induced in 6 to 8 week old female C57BL/6 mice purchased from Charles-River (Italy). Mice were randomly assigned to standard cages, with four to five animals per cage, and kept under standard housing conditions with a light/dark cycle of 12 h and free access to food and water. After 1 week of acclimatization, mice were injected subcutaneously at the flanks with 200 µg of myelin oligodendrocyte glycoprotein p35–55 (MOG35–55) emulsion for the induction of EAE by active immunization. The emulsion was prepared under sterile conditions using MOG35–55 (>85% purity, Espikem, Florence, Italy) in complete Freund’s adjuvant (CFA, Difco), and Mycobacterium tuberculosis H37Ra (8 mg/ml; strain H37Ra, Difco, Lawrence, KS, USA) emulsified with phosphate buffered saline (PBS). The control emulsion was prepared in the same way without MOG35–55 for the control group (CFA group). All animals were injected with 500 ng of pertussis toxin (Sigma, St. Louis, MO, USA) intravenously on the day of immunization and 2 days later according to standard protocols of EAE induction. Animals were scored daily for clinical symptoms of EAE, according to the following scale: 0, no clinical signs; 1, flaccid tail; 2, hind limb weakness; 3, hind limb paresis; 4, complete bilateral hind limb paralysis; 5, death due to EAE; intermediate clinical signs were scored adding 0.5 value [9]–[12].

### Preparation and Activation of BV2 Microglia Cell Line

The BV2 immortalized murine microglial cell line was provided by Dr. F. Aloisi (Department of Cell Biology and Neuroscience, Istituto Superiore di Sanità, Rome, Italy). Briefly, BV2 cells were cultured in DMEM supplemented with 5%F BS, 100 U/ml penicillin and 100 µg/ml streptomycin, and were maintained in a humidified incubator with 5% CO2. 1×10^6^ cells were plated onto 35 mm cell culture dish and treated for 24 h with Th1-specific proinflammatory cytokines [100 U/ml IL-1β (Euroclone), 200 U/ml tumor necrosis factor α (TNFα) (Peprotech), and 500 U/ml interferon γ (IFNγ) (Becton Dickinson) (Th1 mix). For immunofluorescence experiments 2×10^5^ cells were plated onto polylysine coverslips and activated as described. For electrophysiological recordings, BV2 cells were harvested and placed onto a single slice (20 – 30 min).

### Extracellular Recordings

Slices from mouse hippocampus (250–400 µm) were cut in ice-cold ACSF composed of (in mM): NaCl (124), KCl (3.0), MgCl_2_ (1.0), CaCl_2_ (2.0), NaH_2_PO_4_ (1.25), NaHCO_3_ (26), glucose (10); saturated with 95% O_2_, 5% CO_2_, using standard procedures. After an incubation period of 1 h in ACSF at 33.5 °C, the slices were maintained at room temperature or transferred to the recording chamber. For extracellular recordings, the recording electrodes (1.5–2 MΩ; 3 M NaCl) were placed in the *stratum radiatum*. The tungsten bipolar electrode was placed in the *stratum radiatum*, and the Schaffer collateral/commissural fibers were stimulated at 0.033 Hz. For LTP or LTD experiments, the stimulus strength was adjusted to evoke field excitatory postsynaptic potentials (fEPSPs) at approximately 30–40% of the maximal fEPSP amplitude in the Schaffer collateral pathway. Results are reported as mean ± S.E.M. Statistical significance was evaluated by Student’s t-test between 50–60 min following delivery of conditioning trains. Statistical significance was set at *p*<0.05. All experiments and the analysis of data were performed in a blind manner. For all statistical comparisons, the *n* used was the number of animals rather than number of slices.

### Patch-clamp Recordings

For patch-clamp recordings CA1 pyramidal neurons were recorded in whole-cell configuration using 1.5 mm borosilicate glass electrodes (3–4 MΩ) in voltage-clamp mode. Two types of filling solutions were used: 1) for spontaneous or evoked excitatory postsynaptic currents (EPSCs), containing (in mM) CsCl (135), KCl (10), CaCl_2_ (0.05), EGTA (0.1), Hepes (10), Na_3_-GTP (0.3), Mg-GTP (4.0), pH adjusted to 7.3 with CsOH; 2) for spontaneous inhibitory postsynaptic currents (sIPSCs), containing (in mM) KCl (145), CaCl_2_ (0.05), EGTA (0.1), Hepes (10), Na_3_-GTP (0.3), Mg-GTP (4.0), pH adjusted to 7.3 with KOH. Unless otherwise stated, neurons were held at -70 mV. Current signals were filtered at 3 kHz and digitized at 10 kHz using a Multiclamp 700B operated by the pClamp10 software (Molecular Devices, Sunnyvale, CA, USA). No series resistance compensation was implemented, in order to keep a low signal-to-noise ratio, however, recordings were discarded if series resistance changed by more than 15% from control. The spontaneous events were detected from 3 min trace records and analyzed with Clampfit (Molecular Devices, Sunnyvale, CA, USA).

The AMPA/NMDA ratio was obtained from EPSCs elicited at 0.033 Hz with a glass pipette filled with ACSF, close to the dendritic region of the recorded neuron. The AMPA component was evaluated by the peak amplitude of the EPSC recorded at −80 mV holding potential, while the NMDA component in a 2 ms window at 60 ms delay from the stimulation artifact, while holding the cell at +40 mV. Changes in amplitude or inter-event interval of the spontaneous events were compared according to their cumulative distributions, using the Kolmogorov-Smirnov (K-S) test, or their median values, using the Student’s t-test.

### Multi-electrode Recordings

For multi-electrode recordings, individual slices were submerged in ACSF (6 ml/min; 30°C), placed over an 8×8 array of planar microelectrodes, each 50×50 µm in size, with an interpolar distance of 150 µm (MED-P5155; Alpha MED Sciences, Kadoma, Japan) in such a way that the CA1 pyramidal layer and *stratum radiatum* covered most of the planar electrodes. Voltage signals were acquired using the MED64 System operated by the Conductor software (Alpha MED Sciences, Kadoma, Japan), digitized at 20 kHz and low-cut filtered at 1.0 Hz. The power spectral density (PSD) was calculated in the voltage signal acquired from one of the recording electrode placed below the CA1 pyramidal layer, where higher amplitude oscillation were detected, using NeuroExplorer (Nex Technologies, Littleton, USA). The signal was down-sampled to 200 Hz and power spectra were calculated for consecutive 2 min epochs. We then calculated the integral of the power spectra within 25 to 45 Hz and of frequencies <15 Hz, at the time when drug-induced gamma oscillations reached their highest amplitude. The two PSD areas were then normalized to those obtained within the same range of frequencies, during the 2 min preceding drug perfusion. Drugs were applied by dissolving them to the desired final concentration in the bathing ACSF and were as follows: Picrotoxin (100 µM), CNQX (10 µM) from Tocris Cookson (Bristol, UK); IL-1β (30 ng/ml) and IL-1 receptor antagonist (IL-1ra; 10 µg/ml) from R&D Systems (Minneapolis, USA).

### Immunofluorescence and Confocal Imaging

Immunohistochemistry, microscopy and image analyses were performed as described previously [13]. Mice were killed at the peak of the acute phase (20 dpi). They were deeply anesthetized with avertine and perfused through the aorta with ice-cold 4% paraformaldehyde. Brains were post-fixed for at least 4 h at 4°C and equilibrated with 30% sucrose overnight (ON). Thirty micrometer-thick sections were permeabilized in PBS with Triton-X 0.25% (TPBS). All following incubations were performed in TPBS. Sections were pre-incubated with 10% normal donkey serum solution for 1 h at room temperature (RT) and incubated with the following primary antibodies: mouse anti-PV (1∶300, Sigma-Aldrich, P3088), rabbit anti-Iba1 (1∶500, Wako, 019-19741), goat anti-IL-1β (1∶200, R&D System, AF-401-NA), ON at +4°C. After being washed for three times (10 min each), sections were incubated with the following secondary antibodies: Alexa-488 conjugated donkey anti-mouse or anti-rabbit (1∶200, Invitrogen) and Cy3-conjugated donkey anti-rabbit or anti-goat (1∶200, Jackson) for 2 h at RT and rinsed (last wash in Dapi 0.01 mg/ml). Sections were mounted with Vectashield (Vector Labs, USA) on poly-L-lysine coated slides, air-dried and coverslipped. Images from immunolabeled samples were acquired with a Zeiss LSM700 confocal imaging system using 5×, 20×, 63× objectives (pixel resolution 1024×1024, digital zoom 0.5×). For quantitative analysis a 10× objective (pixel resolution 1024×1024, digital zoom 0.8×) was used to acquire anti-PV immunostaining stacks of the dorsal hippocampus from coronal sections. Subtraction of the background and smooth filter were applied on stacks to reduce noise by NIH ImageJ software (http://rsb.info.nih.gov/ij/). Each image stack was z-projected and exported in TIFF file format and PV-positive neurons were counted manually on the z-projections and divided by the area covered by the CA1 and *stratum oriens* of the hippocampus. Measurements were performed on both left and right hippocampus (n = 3 mice per group) from 4 to 5 sections per animal.

Immunocytochemistry: activated BV2 cells and the relative control were washed twice with PBS and fixed in PBS containing 4% (w/v) paraformaldehyde for 15 min at room temperature. Cells were washed with PBS 1X pH 7.4 and permeabilized with 0.1% (v/ v) Triton X-100/PBS pH 7.4 for 4 min at room temperature. Coverslips were saturated with 2% BSA and 10% FCS in PBS 1X for 3 h followed by incubation overnight at 4 °C in a humidified chamber with primary antibody. Unbound antibody was removed by three washes with PBS 1X. Bound antibody was detected by incubation with donkey anti-goat Alexafluor-488 conjugated secondary antibody (Jackson, dil 1∶200) for 60 min. Nuclei were stained with DAPI (Sigma, St. Louis, MO, U.S.A.) for 5 min. Goat anti-interleukin-1β (1∶200, R&D System, AF-401-NA) was used as primary antibody. Images were acquired with a Zeiss LSM700 confocal imaging system using 63× objectives (pixel resolution 1024×1024, digital zoom 1×).

## Results

### Aberrant Plasticity in the Hippocampus of EAE Mice and in Response to IL-1β

**Figure 1:**
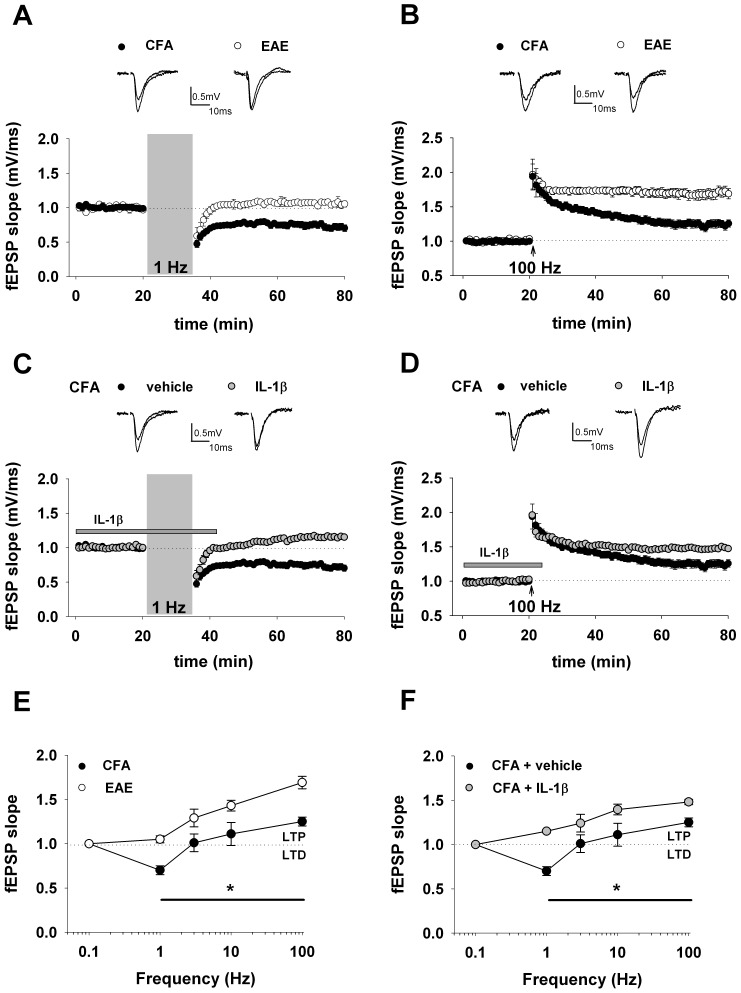
Shifted frequency-response curve in the hippocampus of EAE mice and in response to IL-1β. A) Sample traces (above) and summary graph (below) of the averaged time course of LTD (CFA, n = 11; EAE, n = 12) induced by paired-pulse low-frequency stimulation protocol (PP-LFS: 1 Hz, 15 min). Here and after, insets show field EPSPs from representative experiments during a baseline interval and 60 min after delivery of conditioning train. B) Sample traces (above) and summary graph (below) of the averaged time course of LTP (CFA, n = 9; EAE, n = 12) induced by high-frequency stimulation (HFS: 100 Hz; 1 s). C) Sample traces (above) and summary graph (below) of the averaged time course of LTD induced by PP-LFS in CFA animals with (n = 10) or without (n = 11) IL-1β application (30 ng/ml). Bar indicates the exposure time to IL-1β. D) Sample traces (above) and summary graph (below) of the averaged time course of LTP induced by HFS in CFA animals with (n = 9) or without (n = 9) IL-1β application. E) Frequency-response function in EAE and CFA mice. The graph shows the percentage change in synaptic strength from baseline in EAE and CFA animals at 60 min following stimulation at the indicated frequencies (at least 7 slices were tested for each condition). 0.1 Hz was used to monitor synaptic transmission throughout the experiments. Values are mean ± S.E.M. * indicates *p*<0.05 (t-test) at all frequency tested. F) Shown is a frequency–response graph of the fEPSP changes induced by four different stimulus frequencies in CFA animals with or without IL-1β.

To provide a possible synaptic substrate of EAE-induced learning deficits [14], we investigated bidirectional synaptic plasticity at CA3–CA1 synapses in hippocampal slices from EAE mice (20–25 dpi) and from relative CFA controls. As first predicted on theoretical background [15] and later confirmed experimentally on hippocampal slices [16], low-frequency stimulation produces LTD, intermediate frequencies produce either no change or a low level of LTP, and high-frequency causes LTP. Notably, paired-pulse low-frequency stimulation (PP-LFS) at 1 Hz for 15 min, a protocol known to induce LTD in adult rodents [17], triggered LTD only in CFA slices (n = 11), whereas synaptic responses were potentiated slightly above baseline in EAE slices (n = 12) following the same protocol (CFA, 0.7 ± 0.05; EAE, 1.05 ± 0.04; *p*<0.05) (Fig. 1A).

At a stimulation frequency of 100 Hz (1 s), LTP was induced in both EAE (n = 12) and CFA (n = 9) slices. However, LTP levels were significantly enhanced in EAE slices compared to CFA controls (CFA, 1.25 ± 0.05; EAE, 1.69 ± 0.07; *p<*0.05) (Fig. 1B).

To examine the mechanism underlying the shift in the frequency–synaptic response function associated with EAE, we first pre-incubated and then perfused hippocampal slices from CFA mice with IL-1β (30 ng/ml). Whereas no effects on baseline transmission were observed in the presence of IL-1β, the LTD induced in control conditions by PP-LFS was replaced by a delayed moderate long-lasting potentiation of synaptic transmission (ctrl, 0.7 ± 0.05, n = 10; IL-1β, 1.15 ± 0.02, n = 11; *p<*0.05) (Fig. 1C). Along the same line, high-frequency stimulation (100 Hz, 1 s) elicited a more robust LTP in CFA slices incubated with IL-1β as compared to controls (ctrl, 1.25 ± 0.05, n = 9; IL-1β, 1.47 ± 0.01, n = 9; *p<*0.05) (Fig. 1D). The effects of different conditioning protocols are summarized in frequency–response plots (Fig. 1E,F). Overall, our *in vitro* data demonstrate that EAE mice are more prone to develop stronger LTP responses than CFA controls (Fig. 1E). Remarkably, incubation of IL-1β was sufficient to mimic the aberrant plasticity observed in EAE mice (Fig. 1F).

### Reduced GABAergic Transmission in the CA1 Hippocampal Area of EAE Mice

**Figure 2:**
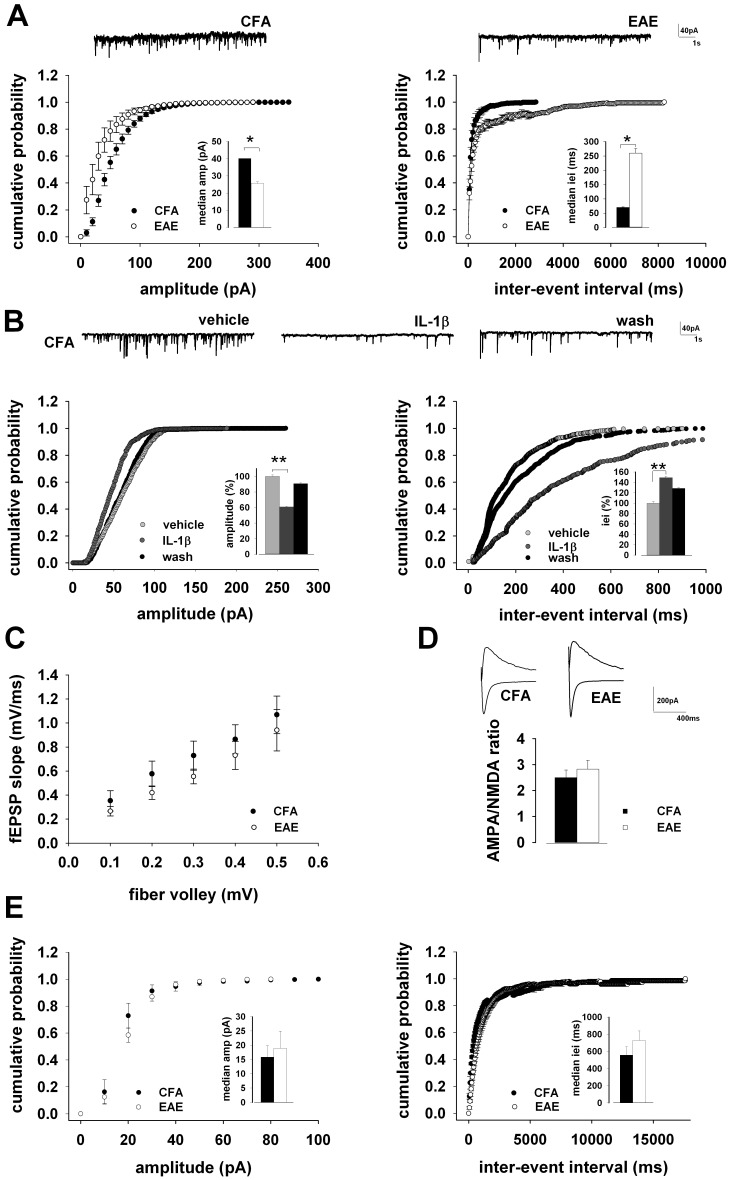
Inhibition of the GABAergic transmission in EAE mice. A) Pooled (mean ± S.E.M) cumulative distributions of sIPSCs amplitude (left; bin size 10 pA) and inter-event interval (right; bin size 50 ms) recorded from neurons of CFA (n = 11) and EAE (n = 9) mice. Histograms in the insets are averages (mean ± S.E.M) of the corresponding median values for neurons of CFA and EAE mice. On top are representative trace records from CFA (left) or EAE (right) mouse. B) Cumulative distributions of sIPSCs amplitude (left) and inter-event interval (right) recorded from a single CFA neuron in response to IL-1β (30 ng/ml). The traces on top are obtained from same neuron, in control conditions, during IL-1β application and at IL-1β washout. Histograms in the insets are averages (mean ± S.E.M; n = 11) of the corresponding median values. C) Input/Output curves (mean ± S.E.M) of fiber volley amplitude vs. fEPSP slope, from extracellular recordings in the CA1 area of 9 slices of CFA and 12 slices of EAE mice. D) Histograms (mean ± S.E.M) of the AMPA/NMDA ratio of evoked EPSCs recorded in whole-cell from 9 neurons of CFA and 13 neurons of EAE mice. On top are representative traces of EPSCs recorded al −80 mV and +40 mV holding potential from CFA (left) and EAE (right) mouse. E) Pooled (mean ± S.E.M) cumulative distributions of sEPSCs amplitude (left; bin size 10 pA) and inter-event interval (right; bin size 50 ms) recorded from neurons of CFA (n = 9) and EAE (n = 13) mice. Histograms in the insets are averages (mean ± S.E.M) of the corresponding median values for neurons of CFA and EAE mice. * and ** indicate *p<*0.05 and 0.01 t-test, respectively.

Both increased glutamatergic transmission and reduced GABAergic transmission has been found in the striatum [10], [18] and cerebellum [13] of EAE mice, and IL-1β replicates to some extent these synaptic alterations [7], [8], [13]. If analogous synaptic abnormalities occur also in the hippocampus, this could explain why the threshold for LTP induction is reduced in EAE. We therefore explored possible differences in the efficacy of the GABAergic and glutamatergic transmission in CA1 pyramidal neurons of EAE compared to CFA mice. We recorded sIPSCs from 11 neurons of CFA mice and from 9 neurons of EAE mice (20–25 dpi), in the continuous presence of the AMPA receptor antagonist CNQX (10 µM). We found a reduced efficacy of the GABAergic transmission in EAE mice, expressed by sIPSCs of reduced amplitude and larger inter-event interval (Fig. 2A).

**Figure 3:**
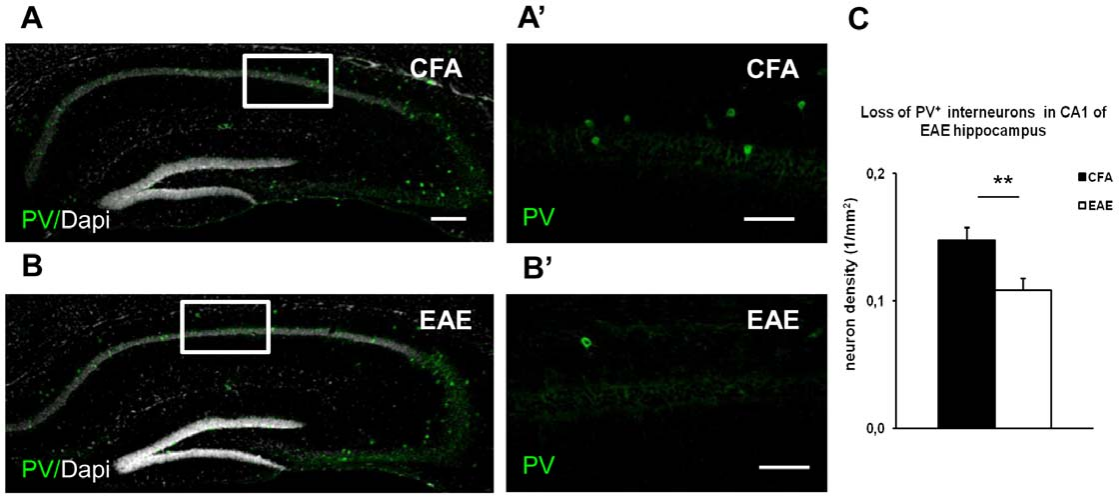
Loss of PV^+^ interneurons in the EAE hippocampus. (A–C) Immunostaining of PV^+^ neurons (green) in hippocampal coronal sections derived from CFA (A) and EAE mice at 20 dpi (B), respectively. The hippocampal structure is defined by fluorescent signal derived from DAPI positive cell nuclei (gray). A loss of PV^+^ neurons characterizes EAE mice. A’ and B’ are high magnifications of the white boxes in A and B panels, respectively. The number of PV^+^ interneurons (lower panels) in the CA1 layer and *stratum oriens* was reduced in EAE mice relative CFA mice. C) Histogram shows the mean percentage of the PV^+^ density interneurons (1/mm^2^) which was significantly reduced in EAE mice by about 27% relative to CFA in the acute phase of the disease. ** indicates *p<*0.01, t- test. Scale bar 200 µm in A and B; 100 µm in A’ and B’.

This effect was proven by the leftward shift of the cumulative sIPSCs amplitude distribution (*p<*0.01, K-S test) in neurons from EAE mice and reduction of their median values (*p<*0.05, t-test; Fig. 2A left), combined with a rightward shift of the cumulative inter-event interval distribution of the same events (*p<*0.01, K-S test) and an increase of their median values (*p<*0.05, t-test; Fig. 2A right). The decreased efficacy of GABAergic transmission found in EAE mice could be mimicked by perfusion of IL-1β (30 ng/ml) for 10 min on CFA slices (Fig. 3B). Thus, a reversible leftward shift of the cumulative amplitude distribution was observed in 11 neurons from CFA mice 10 min after the onset of IL-1β perfusion (*p<*0.001, K-S test, control vs. IL-1β), and reduction of their median values (*p<*0.01, t-test, control vs. IL-1β; Fig. 2B left). This effect was associated with a reversible rightward shift of the cumulative inter-event interval distribution of the same events (*p<*0.001, K-S test, control vs. IL-1β) and increase of their median values (*p<*0.01, t-test, control vs. IL-1β; Fig. 2B right).

Hippocampal glutamate transmission has been recently found impaired in another EAE model, when compared to healthy, CFA-untreated, control mice [19]. In our EAE model, however, no significant difference was found in the efficacy of the glutamatergic transmission in the CA1 area of CFA and of EAE mice. Indeed, the input-output curves obtained from fEPSP recordings in CA1 were similar in hippocampal slices from CFA (n = 9) and EAE mice (n = 12) (Fig. 2C). Moreover, no difference was found in NMDA receptor contribution to CA3-CA1 excitatory synapses in neurons of EAE and CFA mice, evaluated by the AMPA/NMDA ratio (see methods), in the continuous presence of the GABA_A_ receptor antagonist picrotoxin (100 µM; Fig. 2D). No significant difference was found in spontaneous EPSCs amplitude or frequency, in 8 neurons of CFA and 11 neurons of EAE mice recorded in whole-cell configuration in the presence of picrotoxin (100 µM) (Fig. 2E).

### EAE is Associated with Selective Neurodegeneration of PV^+^ Neurons

To support our neurophysiological findings, we explored whether the number of PV^+^ GABAergic neurons was reduced in EAE hippocampus under our experimental conditions. We performed immunohistochemistry and confocal imaging to analyze the density of PV^+^ inhibitory interneurons in the hippocampi derived from CFA and EAE mice at 20 dpi (Fig. 3A–B). The density of PV^+^ neurons was lower in the hippocampus of EAE mice as compared to the hippocampus of CFA mice (Fig. 3A’,B’). In particular, the density of PV+ neurons was reduced by about 27% in the CA1 layer and *stratum oriens* of EAE mice (EAE = 0.11 neurons/mm^2^; control = 0.15 neurons/mm^2^; n = 3 mice, respectively; *p<*0.01) (Fig. 3C). These results are in accordance with Ziehn and colleagues (2010) [14], who performed similar experiments in EAE male mice in comparison with healthy controls. Interestingly, a reduction of PV^+^ inhibitory interneurons has also been shown in the striatum and cerebellum of EAE mice [13], [18], suggesting a selective vulnerability of this neuronal population to neuroinflammation associated with EAE.

### Boosting GABAergic Transmission does not Rescue sIPSC Deficits in EAE

To better understand the relevance of GABA neuron loss in GABAergic transmission deficits found in EAE mice, we performed experiments in the presence of high concentrations of forskolin (30 µM). Forskolin, in fact, enhances synaptic transmission by saturating the evoked and spontaneous release of GABA from hippocampal interneurons [20].

**Figure 4:**
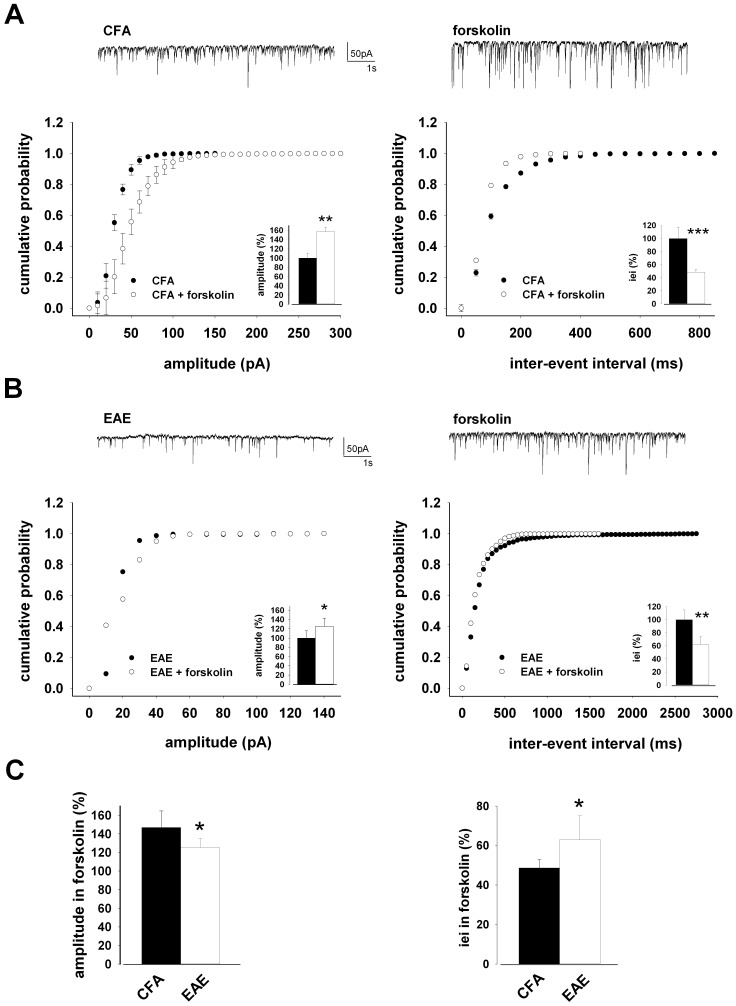
GABAergic transmission is lower in EAE mice in the presence of forskolin. A) Pooled (mean ± S.E.M) cumulative distributions of sIPSCs amplitude (left; bin size 10 pA) and inter-event interval (right; bin size 50 ms) recorded from neurons of CFA in the absence and presence of forskolin (30 µM). Histograms in the insets are averages (mean ± S.E.M) of the corresponding median values for neurons of CFA, in control conditions and following forskolin perfusion. On top are representative trace records from CFA (left) or CFA in the presence of forskolin (right). B) Cumulative distributions of sIPSCs amplitude (left) and inter-event interval (right) recorded from EAE slices in control conditions and in response to forskolin. The traces on top are obtained from same neuron, in control conditions and during forskolin application. Histograms in the insets are averages (mean ± S.E.M) of the corresponding median values. C) Histograms (mean ± S.E.M) of the maximal effect on sIPSC amplitude (left) and inter-event interval (right) reached in the presence of forskolin both in CFA (n = 10) and EAE (n = 14) mice.

**Figure 5:**
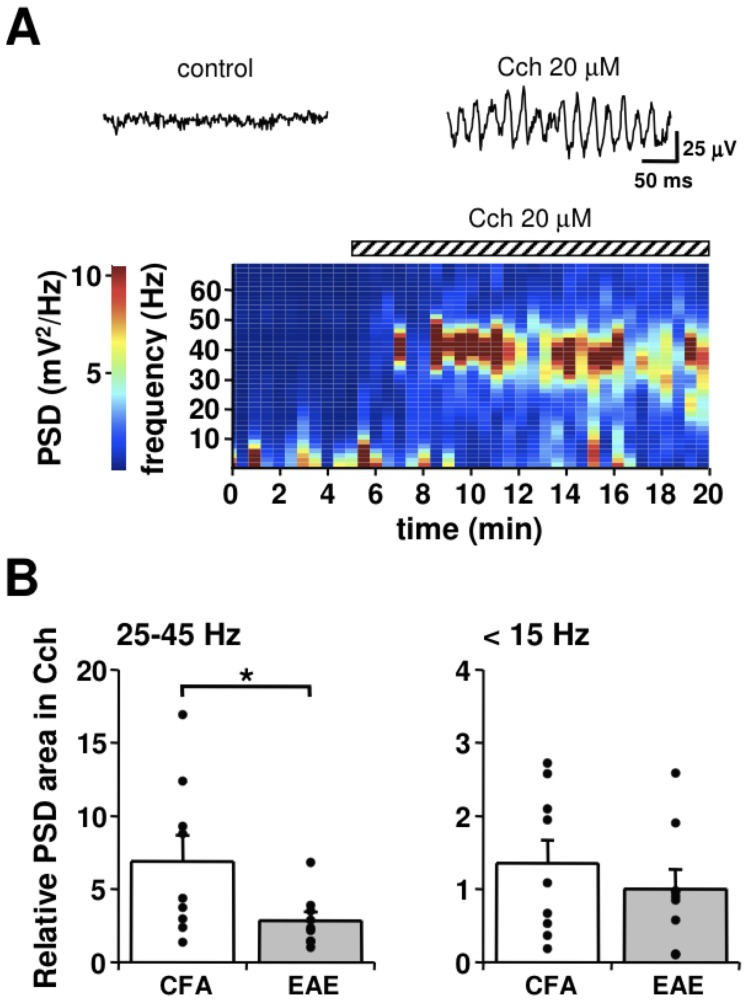
Reduced gamma oscillations in hippocampal slices of EAE mice. A) Spectrogram against time, showing the increase of PSD in the frequency range of 30 to 50 Hz, induced by Cch (20 µM) in the CA1 area of a CFC mouse hippocampal slice. PSD is expressed with a colorimetric scale, measured in successive 1 min epochs. The traces on top are local field potentials recorded in the CA1 pyramidal layer, before (left) and during (right) Cch perfusion. B) Histograms (mean ± S.E.M) of the change in PSD area in Cch (20 µM), for the range of frequencies indicated on top, relative to corresponding measurements obtained before Cch perfusion, in slices from CFC (n = 9) and EAE (n = 9) mice. The scattered dots on each column are single experimental data. * indicates *p<*0.05, t-test.

Perfusion of hippocampal slices with forskolin increased amplitude of GABAergic transmission in CFA mice, as demonstrated by the reversible rightward shift of the cumulative amplitude distribution observed in 10 neurons from CFA mice after the onset of forskolin application (*p<*0.001, K-S test, control vs. forskolin), and increase of their median values (*p<*0.01, t-test, control vs. forskolin; Fig. 4A left). This effect was associated with a reversible leftward shift of the cumulative inter-event interval distribution of the same events (*p<*0.001, K-S test, control vs. forskolin) and decrease of their median values (*p<*0.001, t-test, control vs. forskolin; Fig. 4A right). Although to a lesser extent, also EAE mice displayed an enhanced sIPSC amplitude (*p<*0.001, K-S test on the cumulative distributions; *p<*0.05, t-test on the median values; Fig. 4B left) and smaller inter-event interval (*p<*0.001, K-S test on the cumulative distributions; *p<*0.01, t-test on the median values; Fig. 5A right) observed in 14 neurons following forskolin application. However, the maximal effect reached was always lower in EAE mice compared to CFA controls (*p<*0.05, t-test; Fig. 4C), suggesting that loss of GABAergic nerve contacts significantly contributes to the alterations of sIPSCs in EAE hippocampal neurons.

### Reduced Gamma-frequency Oscillations in the Hippocampus of EAE Mice

It is known that the hippocampus generates gamma-oscillations involved in memory storage and retrieval [21], driven by local PV^+^ GABAergic interneurons [22]–[25]. Therefore, we investigated whether the reduced GABAergic inhibition observed in EAE mice might cause modifications in hippocampal gamma oscillations, induced by perfusion of hippocampal slices with the cholinergic agonist carbachol (Cch) [26]. Oscillations of the local field potential consistently developed in the CA1 area (Fig. 5A) within few minutes of Cch, (20 µM) perfusion, at a frequency range of 20 to 40 Hz. This range is lower than that normally observed *in vivo*, because of the lower temperature of our recording conditions, which allows gaining a better signal-to-noise ratio [27], [28]. The power spectral density (PSD) in the gamma frequency generated in the presence of Cch was then compared in CFA and EAE mice in the acute phase (20–25 dpi). To this aim, we integrated the raw PSD values measured during the maximal expression of Cch-induced local field oscillations (normally after 5 to 10 min of Cch) within two ranges of frequency: 25 to 45 Hz, where gamma oscillations are maximally expressed, and frequencies <15 Hz, where the contribution of gamma oscillations is absent or negligible. These values were then normalized to the integrated PSD, calculated in the same frequency range before Cch perfusion. As shown in Fig. 5B, the PSD within the gamma frequency range was significantly lower in slices from EAE mice (*p<*0.05, t-test), while no significant difference was found in the frequency range <15 Hz.

### Role of Infiltrating Microglia/Macrophages in Hippocampal Synaptic Alterations

**Figure 6:**
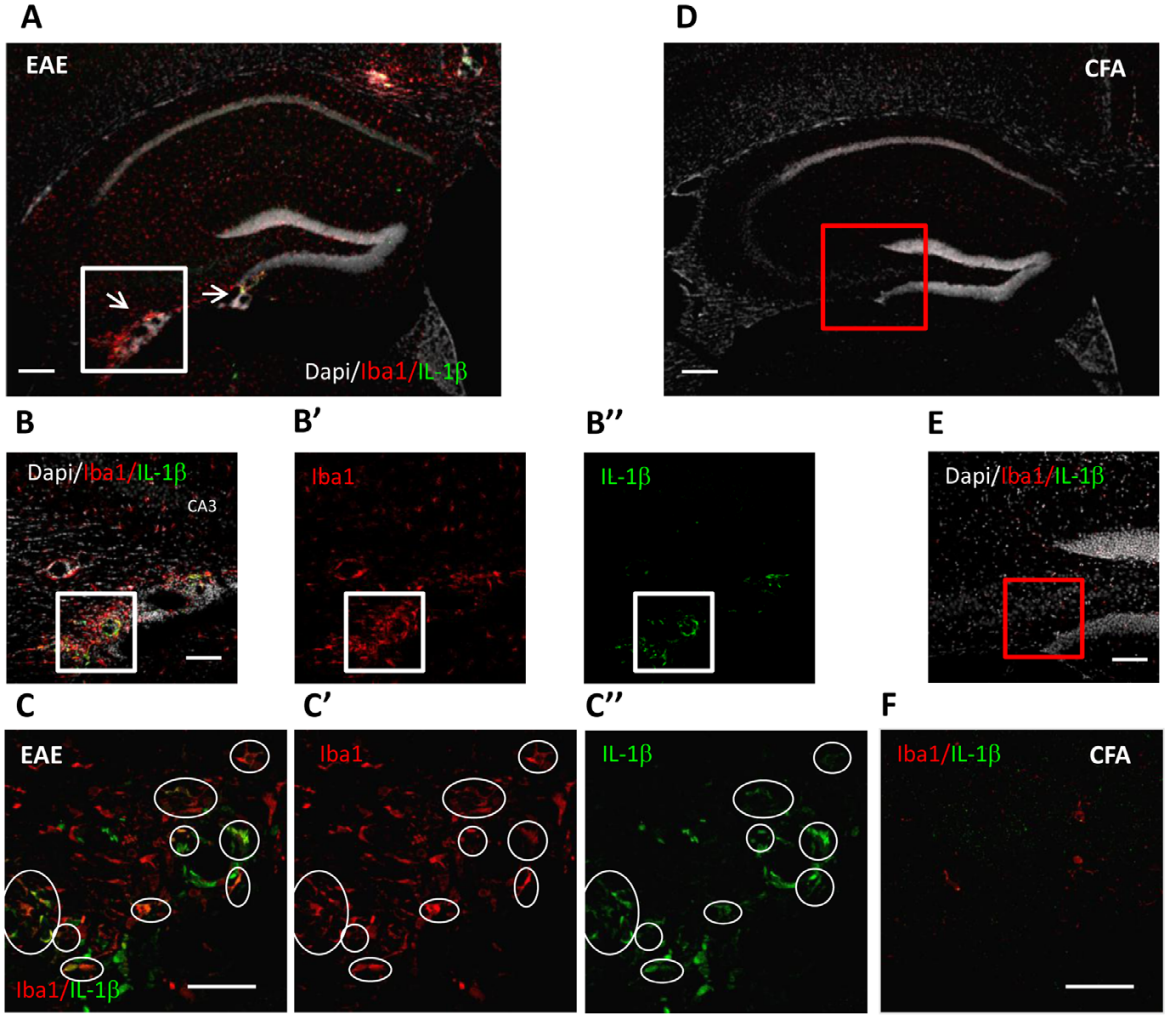
Subsets of activated microglia/macrophage in EAE hippocampus express IL-1β. Double immunostaining of hippocampal coronal sections showing expression of IL-1β (green) in Iba1-positive microglia/macrophage cells (red), which was strongly activated in EAE mice (A–C) (20 dpi; score ≥2) but not in CFA mice (D–F). Arrowheads in A show the lesion sites inside the EAE hippocampus. B–C) In the EAE lesion sites marked out by DAPI staining (B) and localized in proximity of the dentate gyrus, CA3 and fimbria, a colocalization between IL-1β (B”) and Iba1 (B’) was evident (merge image in B), indicating a strong expression of the cytokine in these cells. No lesions and colocalization signals were detected in CFA hippocampus (E–F). B and E are high magnifications of the boxes in A (white box) and D (red box), respectively. On the contrary, the activated microglia cells (B’) which were far from the lesion sites did not to express detectable levels of IL-1β (B”). C and F are high magnifications of the white boxes in B and F, respectively. Scale bars in A and D is 200 µm; B–B’–B” and E is 100 µm; in C–C’–C” and F is 50 µm.

Our results indicate that not only GABAergic neuronal loss but also IL-1β might contribute to down-regulate inhibitory synaptic transmission during EAE. Thus to confirm this hypothesis, we decided to investigate the expression of the IL-1β in the hippocampus of EAE mice at the peak of the disease. Since potential source of IL-1β are microglia and macrophages, which are strongly activated (Iba1-positive) at this stage of the disease, we performed double immunofluorescence experiments and confocal imaging on hippocampal coronal slices derived from EAE (20pi) in comparison to CFA mice (Fig. 6). Besides the presence of microglia/macrophages broadly activated in all EAE hippocampus (Fig. 6A), we observed a strong Iba1-positive labeling in EAE-induced lesion sites (DAPI-positive, Fig. 6A–B) localized in proximity of the dentate gyrus, CA3 region and fimbria. Of note, IL-1β staining (Fig. 6B–B”) was clearly detectable in the lesion sites enriched of activated microglia/macrophages (B’), while it was virtually absent in the rest of the hippocampus likely due do the limitations of the immunofluorescence technique. However, a differential expression of IL-1β among different microglia/macrophages populations is plausible [29]. In the lesion site we indeed observed a variability of IL-1β expression in Iba1-positive cells including no colocalization (Fig 6C–C’–C”). Notably, other sources of IL-1β, different from microglia/macrophages, were also present in the EAE hippocampus. On the contrary, the expression of IL-1β was not detected in the CFA hippocampus where lesions sites and activated microglia/macrophages were absent (Fig. 6 D–E–F).

### Activated Microglia Alter GABA Transmission in an IL-1β-dependent Manner

**Figure 7:**
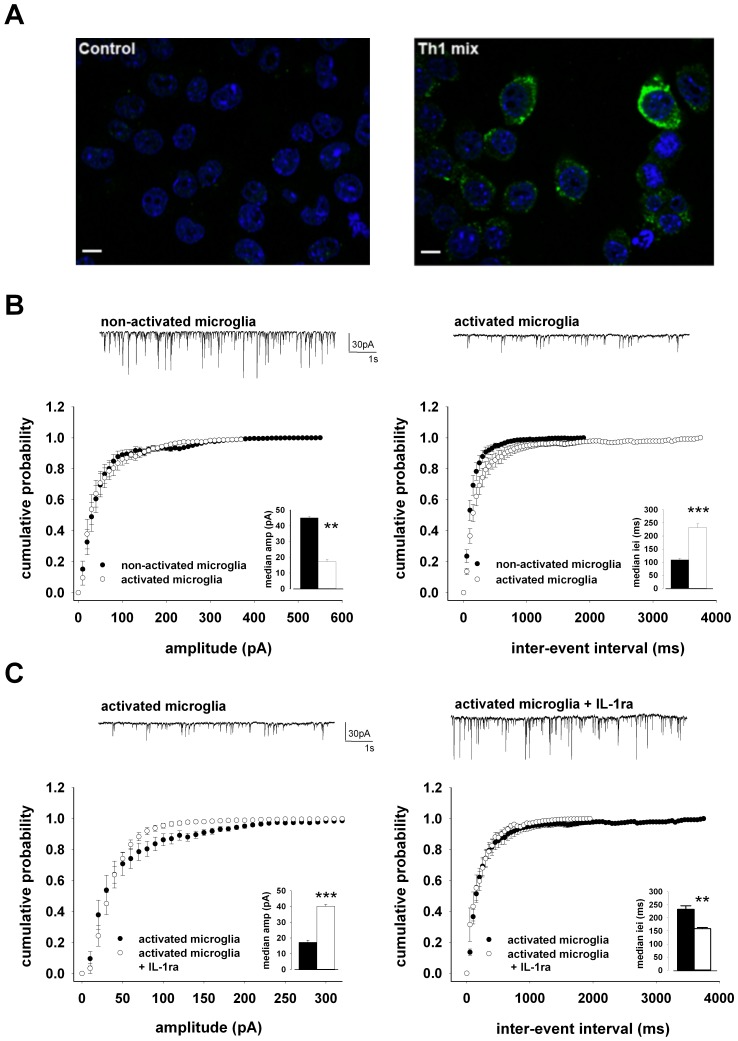
IL-1β-dependent inhibition of the GABAergic transmission by activated microglia. A) Representative image of immunostaining for IL-1β (green) on BV2 microglial cells (blue signal from DAPI) in basal condition (control) and after activation with Th1 pro-inflammatory cytokine mix. Control cells do not exhibit IL-1β staining, while the expression of the cytokine is visible in activated cells. Scale bar: 10 µm. B) Pooled (mean ± S.E.M) cumulative distributions of sIPSCs amplitude (left; bin size 10 pA) and inter-event interval (right; bin size 50 ms) recorded from neurons of C57BL/6 mice pre-incubated in non-activated (n = 12) and activated microglia (n = 12). Histograms in the insets are averages (mean ± S.E.M) of the corresponding median values. On top are trace records from typical neurons exposed to non-activated (left) or activated (right) microglia. C) Pooled (mean ± S.E.M) cumulative distributions of sIPSCs amplitude (left; bin size 10 pA) and inter-event interval (right; bin size 50 ms) recorded from neurons of C57BL/6 mice pre-incubated in activated microglia (n = 12) or activated microglia with the IL-1β receptor antagonist IL-1ra (10 µg/ml; n = 12). Histograms in the insets are averages (mean ± S.E.M) of the corresponding median values. On top are trace records from typical neurons exposed to activated microglia (left) or activated microglia with IL-1ra (right). ** and *** indicate *p<*0.01 and 0.001 t-test, respectively.

The observation that microglial activation was associated with IL-1β staining in EAE hippocampus led us to hypothesize that microglia could contribute to sIPSC deficits by producing and releasing IL-1β [10], [13]. To assess the potential involvement of microglia in the synaptic alterations found in EAE mice, we pre-incubated (20–30 min) hippocampal slices from C57BL/6 mice in the presence of activated BV2 microglial cells, and recorded sIPSCs from CA1 pyramidal neurons in the continuous presence of CNQX (10 µM). As we observed in activated microglia in EAE hippocampi, also BV2 microglial cells expressed IL-1β upon activation with a Th1 cytokine mix (Fig. 7A). In 12 slices pre-incubated with activated microglia [10], we found a reduced efficacy of GABAergic transmission compared to 12 slices incubated with non-activated microglia. Accordingly, we found a reduced sIPSC amplitude (*p<*0.001, K-S test on the cumulative distributions; *p<*0.01, t-test on the median values; Fig. 7B left) and larger inter-event interval (*p<*0.001, K-S test on the cumulative distributions; *p<*0.001, t-test on the median values; Fig. 7B right) caused by activated microglia, reminiscent of the alterations seen in EAE slices or in response to IL-1β. The decrease in the efficacy of GABAergic transmission by activated microglia was prevented by co-incubation with the IL-1β receptor antagonist IL-1ra (10 µg/ml) (n = 12; Fig. 7C). In fact, in the presence of IL-1ra, we recorded sIPSCs of higher amplitude (*p<*0.001, K-S test on the cumulative distributions; *p<*0.001, t-test on the median values) and lower inter-event interval (*p<*0.01, K-S test on the cumulative distributions; *p<*0.01, t-test on the median values).

## Discussion

Almost half of MS patients develop some degree of cognitive dysfunction during their lifetime, with a prevailing pattern of attentional, memory, executive function, and visuo-spatial deficits [6], [30], [31]. The pathophysiology of cognitive deficits in MS has been largely unexplored. In this study, we showed a profound subversion of plasticity rules and mechanisms in the EAE hippocampus, which is likely caused by the inflammatory milieu generated by infiltrating autoreactive T lymphocytes in the CNS. In this respect, it has been postulated that repetitive synaptic stimulation induces a mixture of excitatory and inhibitory long-lasting effects, the direction of which being determined in physiological conditions by the excitation/inhibition balance [32]. Thus, the increased glutamate transmission [7] and reduced GABA inhibition [8, present study] found in EAE brains are likely to play a significant role in the observed LTD deficits. In line with this, implication of GABA in the generation of LTD phenomena in the neocortex has been observed in animals [33]–[35], and results of the present investigation contribute to assign to GABAergic transmission a crucial role in LTD induction over LTP in the hippocampus.

The threshold for the induction of synaptic plasticity in the brain does not rely only upon the NMDA receptor, but rather depends on the complex interplay between excitatory and inhibitory neurotransmitters. For example, attenuation of LTP by GABA_A_ receptor agonists, particularly when slices are stimulated in the theta frequency range, has been generally attributed to phasic inhibitory currents generated by synaptic GABA_A_ receptors [36]–[42]. Other studies also showed that low-to-moderate concentrations of GABA_B_ receptor antagonists are able to facilitate LTP [33], [43], [44]. On the other hand, elevating GABAergic activity by pharmacological blockade or genetic deletion of GABA transporter-1 (GAT1), the protein that removes synaptically released GABA, specifically impairs LTP, and affects hippocampal theta-oscillation and hippocampus-dependent learning and memory [45].

Both increased glutamatergic transmission and reduced GABAergic transmission have been found in the striatum [10], [18] and cerebellum [13] of EAE mice. To provide a reliable explanation of the plasticity defects seen in EAE brains, we therefore explored possible differences in the efficacy of the GABAergic and glutamatergic transmission also in CA1 pyramidal neurons of EAE and CFA mice. We found that spontaneous release of GABA was reduced in EAE mice, indicating a reduced efficacy of the GABAergic transmission. Conversely the glutamatergic transmission was unaltered, both in terms of spontaneous release and AMPA/NMDA ratio. These findings may well explain the rightward shift in the frequency–synaptic response function observed in EAE mice, and are reminiscent of the alterations leading to plasticity subversion in MS patients.

To better understand the potential cellular mechanisms supporting our neurophysiological findings, changes in hippocampal neuronal architecture were assessed in the CA1 region of EAE mice. In line with previous observations in the striatum [18], cerebellum [13] and hippocampus [14], here we showed that the number of GABAergic interneurons was reduced in EAE hippocampus, suggesting that selective vulnerability of this neuronal population may contribute to sustain synaptic hyperexcitability in EAE. Notably, these data are in good agreement with previous findings showing selective loss of PV^+^ interneurons, and reduced extension of PV^+^ neurites in the normal appearing grey matter and in the frontal cortex of MS patients [46], [47]. A defective cortical GABAergic transmission has been postulated in MS patients on the basis of neurophysiological findings with paired-pulse transcranial magnetic stimulation [48], and in experiments showing that the CSF from active MS patients is able to inhibit GABAergic sIPSCs recorded in mouse brain slices [8].

Local PV^+^ GABAergic interneurons heavily regulate gamma-oscillations in the hippocampus, playing a key role in the synchronization of pyramidal cell firing [22]–[25]. Brain network oscillations in the gamma frequency (30 to 80 Hz) are involved in cognitive processes, representing a further neurophysiological correlate of hippocampus-dependent cognitive abilities [21], [49]–[52]. Thus, we hypothesized that degeneration of PV^+^ neurons could also modify hippocampal gamma frequency, thereby exacerbating the functional consequences of LTD loss in EAE mice. Indeed, besides aberrant synaptic transmission and plasticity, EAE slices also displayed reduced hippocampal gamma oscillations.

We believe that abnormal plasticity and the changes in synaptic function and network activity in the hippocampus of EAE mice largely rely on IL-1β-mediated suppression of GABAergic activity, at least before the degeneration of GABAergic interneurons is established. It is interesting to note that IL-1β increases during focal inflammation in MS at a concentration high enough to influence in opposite directions glutamate transmission (which is enhanced by this cytokine) and GABA synapses (which are instead inhibited) [7], [8]. Not only in MS, but also in EAE, IL-1β plays a substantial role in the inhibition of GABA transmission in the striatum [18] and cerebellum [13]. Consistently, here we showed that IL-1β was sufficient to mimic in the hippocampus of CFA mice the synaptic alterations found in EAE.

Previous reports have generally found that IL-1β at high concentrations contributes to impairment in synaptic function [53]. On the other hand, low concentrations of IL-1β are likely to play a physiological function in LTP maintenance. Accordingly, several studies have demonstrated that full expression of LTP is prevented in the absence of IL-1β and that IL-1ra can block LTP [54]–[57]. Certainly, not only the concentration of IL-1β, but also other experimental variables such as the incubation and perfusion time, the species and age of the animal used, and probably other factors, may contribute to determine the diverse effects of this cytokine on synaptic function. Notably, no studies have yet reported the effects of IL-1β on brain slices from CFA mice. In addition, a decreased efficacy of GABAergic transmission was found in hippocampal slices incubated with preparations of activated microglia, being prevented by blockade of IL-1β signaling with IL-1ra. Our findings suggest that this pro-inflammatory cytokine alters the equilibrium between excitation and inhibition shifting the balance towards excitability and in turn leading to synaptic dysfunction.

In conclusion, the present work suggests that soluble mediators released by microglia during neuroinflammatory conditions might contribute to degeneration of GABAergic interneurons which, in turn, influences synaptic function in the hippocampus of EAE mice. Because the hippocampus is a brain region essential to cognition and vulnerable to degeneration, its dysfunction could certainly contribute to the high prevalence of cognitive impairment associated with MS [57] and to the learning deficits in EAE [14], [58].

A better understanding of how neuroinflammation affects neuronal function and synaptic transmission in MS will hopefully lead to development of neuroprotective strategies aimed at preserving neuronal function and integrity.
